# Prevalence rates of metabolic health and body size phenotypes by different criteria and association with insulin resistance in a Maltese Caucasian population

**DOI:** 10.1186/s12902-022-01071-x

**Published:** 2022-06-15

**Authors:** Rachel Agius, Marie Claire Fava, Nikolai Paul Pace, Stephen Fava

**Affiliations:** 1 Faculty of Medicine and Surgery, University of Malta Medical School, Tal-Qroqq, Msida, Malta; 2 Mater Dei Hospital, Triq Dun Karm, Msida, MSD2090 Malta

**Keywords:** Metabolic Health, Insulin Resistance, Body Mass Index (BMI), HOMA-IR

## Abstract

**Introduction:**

Hyperinsulinemia and insulin resistance are known to be associated with increased cardiovascular morbidity and mortality. A metabolically unhealthy phenotype is frequently used as a surrogate marker for insulin resistance. The aims of the current study were to compare the prevalence of the body size phenotypes using different definitions of metabolic health and to investigate which one of them is most strongly associated with insulin resistance in men and women.

**Methods:**

We conducted a cross-sectional study in a middle-aged cohort of Maltese Caucasian non-institutionalized population. Metabolic health was defined using the various currently used definitions.

**Results:**

There were significant differences in the prevalence of body size phenotypes according to the different definitions. We also found significant sex differences in the predictive value of the various definitions of the metabolically unhealthy phenotype to predict insulin resistance. The strongest association was for the definition of having >2 NCEP-ATPIII criteria to characterize the metabolic unhealthy phenotype in women (odds ratio of 19.7). On the other hand, the Aguilar-Salinas *et al.* definition had the strongest association in men (odds ratio of 18.7).

**Conclusions:**

We found large differences in the prevalence of the various body size phenotypes when using different definitions, highlighting the need for having standard criteria. Our data also suggest the need for sex-specific definitions of metabolic health.

## Introduction

Evidence from several epidemiological studies demonstrates that hyperinsulinemia and insulin resistance are associated with an increased risk of both cardiovascular disease and of all-cause cardiovascular and cancer mortality [1–3]. For example, in the Paris Prospective Study, fasting and 2-hour post-load plasma insulin levels were found to be independent predictors of coronary artery disease and death over an 11-year follow-up period [4]. In the Helsinki Policemen Study, patients in the highest quintile of the area under the insulin response curve during an oral glucose tolerance test had higher all-cause mortality at 10 and 22 years [5]. The Diabetes Epidemiology: Collaborative analysis of Diagnostic Criteria in Europe (DECODE) Insulin Study Group reported similar results and confirmed the association between hyperinsulinemia and higher risk of cardiovascular mortality in both males and females [6].

The direct quantification of insulin resistance can be challenging in clinical practice. Therefore, metabolic health phenotypes are frequently used as surrogate indices of insulin resistance. The concept of metabolic health comprises multiple anthropometric and biochemical parameters that are easily ascertained clinically. The metabolically unhealthy state describes individuals with increased insulin resistance [7, 8] and higher cardiovascular risk [9] irrespective of body mass index (BMI). To date, there is no universally accepted definition of what constitutes metabolic health, and several definitions are currently in use (Table 1). These definitions vary with regard to which parameters are employed, their respective cut-offs and in the number of abnormal parameters needed to characterize a subject as being metabolically unhealthy.

**Table 1:** Criteria currently in use to define metabolic health

	NCEP-ATPIII [ 10]	Wildman *et al*. [11]	Doumatey *et al*. [12]	Hamer *et al*. [13]	Aguilar-Salinas *et al*. [14]	Lynch *et al*. [15]	Karelis *et al*. [16]	Harmonisation criteria (Lavie *et al*.) [17]
BP (mmHg)	SBP ≥ 130 or DBP ≥ 85 or on Rx	SBP ≥ 130or DBP ≥ 85 or on Rx	SBP > 130or DBP > 85 or not on Rx	SBP ≥ 130 or DBP ≥ 85 or on Rx	SBP ≥ 140 or DBP < 90 or not on Rx	SBP > 130or DBP > 85 & not on Rx		SBP ≥ 130 or DBP ≥ 85 or on Rx
TG (mmol/L)	≥1.69 mmol/L	≥ 1.70					≥1.70	≥ 1.70
HDL-C (mmol/L)	HDL-C < 1.03 mmol/L in men or < 1.29 mmol/L in women or on Rx	<1.04 in men or < 1.30 on women	<1.03 in men or < 1.29 in women	<1.03 in men or < 1.30 in women	< 1.00		<1.30 & not on Rx	<1.0 in men or < 1.30 in women
TG/HDL ratio						>1.65 in men or > 1.32 in women & not on Rx		
LDL-C (mmol/L)							≥2.60	
T. Chol (mmol/L)							≥5.20	
FPG (mmol/L)	≥ 5.6 or on Rx	≥ 5.55 or on Rx	≤7.0	Presence of diabetes	≥7.0 or on Rx			≥ 5.6 or on Rx
WC (cm)	>102 in men and > 88 in women			>102 in men or > 88 in women				
HOMA-IR		>5.13					<1.95	
hsCRP (ng/L)		>0.1		>3.0				
**No. of criteria required to characterize the metabolically healthy phenotype**	**0, ≤ 1 or ≤ 2**	**<2 of the above**	**None of the above**	**< 2 of the above**	**None of the above**	**None of the above**	**≤ 1 of the above**	**None of the above**

The criteria used by Meigs *et al*. [18] are the same as NCEP-2

*hsCRP* high-sensitivity C-reactive protein, *HDL-C* high density lipoprotein cholesterol, *HOMA-IR* Homeostatic Model Assessment for Insulin Resistance, *LDL-C* Low density lipoprotein cholesterol, *NCEP-ATP111* National Cholesterol Education Program-Adult Treatment Panel III criteria, *Rx* treatment, *TG* triglycerides, *WC* waist index

Obesity (as defined by the BMI) is strongly correlated with insulin resistance [19] and with a pro-atherogenic cardiometabolic profile [20–23]. Increased adiposity is also associated with increased risk of type 2 diabetes [24, 25], and weight loss by lifestyle intervention reduces this risk [26]. Nonetheless, there are some overweight and obese individuals who do not exhibit the typical cardiometabolic abnormalities associated with obesity and are thus referred to as being metabolically healthy overweight/obese (MHOw/O), thereby distinguishing them from the commoner metabolically unhealthy overweight/obese categories (MUHOw/O). Conversely, there are also some normal weight individuals who are metabolically unhealthy (metabolically unhealthy normal weight (MUHNW)), distinguishing them from the commoner metabolically healthy normal weight (MHNW) state [27]. Thus, these four metabolic health phenotypes enable superior stratification of individual cardiometabolic risk than obesity indices alone.

The aims of the current study were to 1) compare the prevalence of body composition phenotypes using different definitions of metabolic health 2) explore sex differences in the relationship of body composition phenotypes to metabolic health and insulin resistance and 3) evaluate which definition of metabolic health is the strongest predictor of insulin resistance. Since there are sex differences in the relationship of anthropometric parameters to metabolic health and insulin resistance [28], we studied males and females separately.

## Methods

### Study subjects

We conducted a cross-sectional study in a middle-aged cohort (41 ± 10 years) of Maltese Caucasian non-institutionalized population using convenience sampling similar to that used in other studies [29]. Individuals with type 1 diabetes, known underlying genetic or endocrine causes of overweight or underweight (apart from controlled thyroid disorders), active malignancy or terminal illness were excluded. Persons who were unable to give their own voluntary informed consent and pregnant females were also excluded. Anthropometric measurements were recorded with the participants dressed in light clothing and without shoes, using validated equipment which was calibrated in accordance with WHO recommendations [30]. Body weight was measured to the nearest 0.1 kg; height and waist circumference were measured to the nearest 0.1 cm. Normal weight was defined as BMI of 18.5–24.9 kg/m^2^; overweight as BMI of 25.0–29.9 kg/m^2^; and obesity as BMI ≥30 kg/m^2^. Obesity was further subcategorized according to WHO cutoffs as class 1 obesity (BMI 30.0–34.9 kg/m^2^), class 2 obesity (BMI of 35–39.9 kg/m^2^) or class 3 obesity (BMI >40.0 kg/m^2^). Waist index (WI) was calculated as the waist circumference (WC) (cm) / 94 for males and WC (cm) / 80 for females [31]. Fasting insulin and high sensitivity CRP (hsCRP) were quantified at baseline by sandwich ELISA (Diagnostic Automation, USA) according to the manufacturer’s instructions and using a Mithras® microplate reader for absorbance determination. Samples were assayed in duplicate using 50 μL of serum.

### Body size definitions

In the study cohort, metabolic health was defined using the various criteria outlined in Table 1. These include the classifications proposed by Wildman *et al*., (National Health and Nutrition Examination Survey of the United States), Doumatey *et al.*, Hamer *et al.*, Lynch *et al.,* Aguilar-Salinas *et al*., Karelis *et al*. and the harmonization criteria proposed by Lavie *et al* [11–17]. In addition, the NCEP-ATPIII cut-offs for the metabolic syndrome were also applied [10], with subjects being classified as metabolically healthy if they met none of these criteria (NCEP-0), if they had a maximum of one abnormal criterion (NCEP-1), and if they had a maximum of two abnormal criteria (NCEP-2). HOMA-IR was used to evaluate insulin resistance using the formula: fasting serum insulin (μU/ml) x fasting plasma glucose (mmol/L) /22.5 [32]. This is a validated measure of insulin resistance [33, 34]. For binary logistic regression, we defined insulin resistance as HOMA-IR of ≥2.5. We chose this cut-off since it has been linked to increased mortality in large population-based studies [34–36]. Ethical approval for the study was granted by the University of Malta Research Ethics Committee (approval code 06/2016; approval date 08/08/2016).

### Statistics

Normality of distribution was assessed by the Kolmogorov-Smirnov test. Since HOMA-IR exhibited a skewed non-normal distribution, the Mann-Whitney test was used to compare HOMA-IR between metabolically healthy and unhealthy individuals. In order to investigate the predictive value of the various definitions of metabolic health in males and females, we performed logistic regression with HOMA-IR ≥ 2.5 as the dependent variable and a metabolic unhealthy phenotype as the independent variable separately for each of the definitions of metabolic health, except for those by Wildman *et al.* and Karelis *et al.* The latter two were not entered in logistic regression analysis since HOMA-IR is one of the criteria they use to define metabolic health. To investigate the association between BMI category and insulin resistance, logistic regression with HOMA-IR ≥ 2.5 as the dependent variable and BMI category (normal, overweight, obese classes I-III) as the independent variable was performed. Finally, we repeated logistic regression with metabolic unhealthy phenotype using each of the above-mentioned definitions as the independent variable adjusted for BMI category. All analyses were performed using IBM SPSS version 26. A *p*-value of <0·05 was considered significant.

## Results

**Fig. 1:**
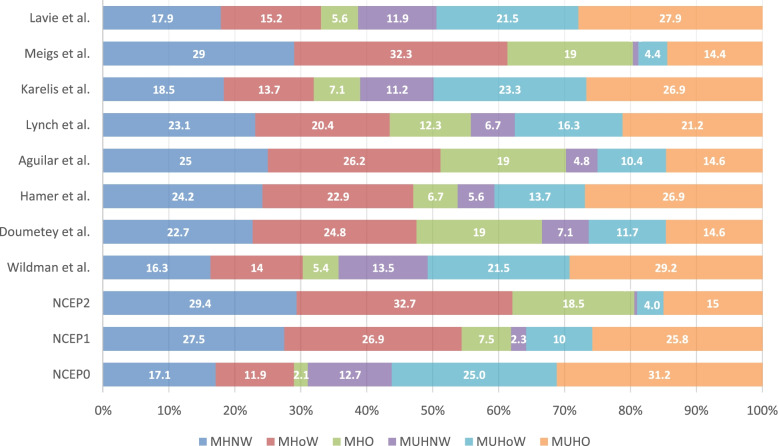
Prevalence (as %) of body size phenotypes using different definitions. NCEP = National Cholesterol Education Program/Adult Treatment Panel III. MHNW = metabolically healthy normal weight; MHoW = metabolically healthy overweight; MHO = metabolically healthy obese; MUHNW = metabolically unhealthy normal weight; MUHoW = metabolically unhealthy overweight; MUHO = metabolically unhealthy obese

We studied 520 individuals of Maltese Caucasian ethnicity (331 females and 190 males). Complete data was obtained for all 520 subjects. The median age in the cohort was 41 years (IQR 6), with a range of 30–51 years. The median age in males was 42 years (IQR 6) and 40 years (IQR 7) in females. The prevalence of the various body size phenotypes according to the different definitions are shown in Fig. 1. The prevalence of metabolically healthy normal weight (MHNW) ranged from 16.3 to 27.5%; metabolically healthy overweight (MHOw) from 11.9 to 32.9%; metabolically healthy obese (MHO) from 2.1 to 19.0%; metabolically unhealthy normal weight (MUHNW) from 0.6 to 13.5%; metabolically unhealthy overweight (MUHOw) from 3.8 to 25.0% and metabolically unhealthy obese (MUHO) from 14.6 to 31.2%.

**Fig. 2:**
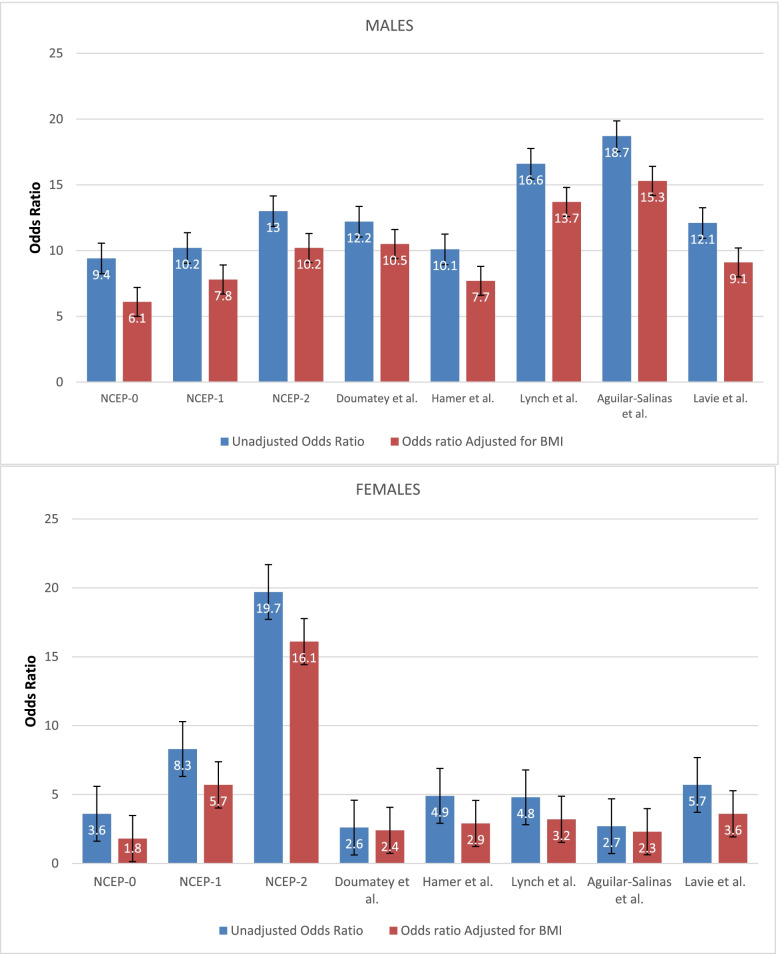
Odds ratio for having a Homeostatic Model Assessment for Insulin Resistance >2.5 in metabolically healthy and unhealthy according to different definitions. Error bars refer to the standard error

In males, a metabolically unhealthy phenotype was associated with a higher median HOMA-IR for all definitions used (*p* < 0.001 for all), as shown in Table 2. In females, a metabolically unhealthy phenotype was also associated with a higher median HOMA-IR for all definitions, except when using the Doumatey *et al.* criteria, as shown in Table 2. Logistic regression analysis revealed that a metabolically unhealthy phenotype was consistently associated with insulin resistance (defined as HOMA-IR ≥ 2.5) across all definitions in both sexes. However, there were notable sex differences in the performance of the various definitions of the metabolically unhealthy phenotype to predict insulin resistance, as evidenced by the odds ratio. This is shown in Table 3 and Fig. 2. In females, the strongest observed association was for the NCEP-2 definition (i.e. having ≤2 NCEP-ATPIII parameters to characterize the metabolically healthy phenotype), with an odds ratio (OR) of 19.7. Conversely, the Doumatey *et al.* criteria had lowest predictive ability in the female cohort (OR of 2.6). In males, the strongest association was for the Aguilar-Salinas *et al.* definition of the metabolically healthy phenotype (OR of 18.7), followed by the Lynch *et al*. (OR of 16.6) and the NCEP-2 (OR of 13.1) definitions (Table 3 and Fig. 2). The Doumatey *et al.* definition performed better in males than in females (OR of 12.2 in males compared to 2.6 in females). When considering BMI category as the sole independent predictor of HOMA-IR ≥2.5, a lower predictive performance relative to a metabolic unhealthy phenotype using any definitions was noted, with OR of 1.90 in females and 2.07 in males (*p* < 0.001).

**Table 2:** Comparison of Homeostatic Model Assessment for Insulin Resistance (HOMA-IR) in the metabolically healthy and unhealthy subgroups as classified by the various definitions and stratified by sex

**Males**			
	**Metabolically healthy**	**Metabolically unhealthy**	***p *value**
	HOMA-IR Median (interquartile range)		
NCEP-0	1.32 (0.89–1.64)	2.12 (1.49–2.71)	<0.001
NCEP-1	1.48 (1.08–1.95)	2.31 (1.90–3.01)	<0.001
NCEP-2	1.61 (1.13–2.19)	2.89 (2.19–3.45)	<0.001
Doumatey *et al.*	1.52 (1.07–1.95)	2.35 (1.95–3.02)	<0.001
Hamer *et al.*	1.51 (1.07–1.95)	2.28 (1.65–2.96)	<0.001
Aguilar-Salinas *et al.*	1.53 (1.08–1.97)	2.51 (1.96–3.08)	<0.001
Karelis *et al.*	1.27 (0.84–1.65)	2.13 (1.53–2.73)	<0.001
Lavie *et al.*	1.29 (0.83–1.66)	2.13 (1.57–2.80)	<0.001
**Females**			
	HOMA-IR Median (interquartile range)		
NCEP-0	1.24 (0.78–1.79)	1.71 (1.09–2.27)	<0.001
NCEP-1	1.31 (0.89–1.84)	2.13 (1.51–2.77)	<0.001
NCEP-2	1.45 (0.95–1.96)	2.72 (2.18–3.19)	<0.001
Doumatey *et al.*	1.52 (0.98–2.07)	1.73 (1.02–2.47)	0.115
Hamer *et al.*	1.36 (0.91–1.85)	1.96 (1.30–2.54)	<0.001
Aguilar-Salinas *et al*.	1.51 (0.96–2.05)	1.88 (1.11–2.66)	0.039
Karelis *et al.*	1.28 (0.82–1.71)	1.97 (1.27–2.55)	<0.001
Lavie *et al.*	1.29 (0.89–1.82)	1.83 (1.20–2.42)	<0.001

*NCEP* National Cholesterol Education Program (NCEP)/Adult Treatment Panel III

**Table 3:** Performance of the various criteria currently in use to define metabolic health to predict insulin resistance, defined as Homeostatic Model Assessment for Insulin Resistance (HOMA-IR) ≥2.5

**Males**		
	**Crude Odds Ratio (*p *value)**	**Odds Ratio (*p *value) adjusted for BMI**
NCEP-0	9.4 (*p* = 0.03)	6.1 (*p* = 0.017)
NCEP-1	10.2 (*p* < 0.001)	7.8 (*p* < 0.001)
NCEP-2	13.1 (*p* < 0.001)	10.2 (*p* < 0.001)
Doumatey *et al.*	12.2 (*p* < 0.001)	10.5 (*p* < 0.001)
Hamer *et al.*	10.1 (*p* < 0.001)	7.7 (*p* < 0.001)
Lynch *et al.*	16.6 (*p* < 0.001)	13.7 (*p* < 0.001)
Aguilar-Salinas *et al.*	18.7 (*p* < 0.001)	15.3 (*p* < 0.001)
Harmonisation criteria (Lavie *et al*.)	12.1 (*p* = 0.001)	9.1 (*p* = 0.003)
**Females**		
NCEP-0	3.6 (*p* = 0.002)	1.8 (*p* = 0.22)
NCEP-1	8.3 (*p* < 0.001)	5.7 (*p* < 0.001)
NCEP-2	19.7(*p* < 0.001)	16.1 (*p* < 0.001)
Doumatey *et al.*	2.6 (*p* = 0.003)	2.4 (*p* = 0.008)
Hamer *et al.*	4.9 (*p* < 0.001)	2.9 (*p* = 0.008)
Lynch *et al.*	4.8 (*p* < 0.001)	3.2 (*p* = 0.001)
Aguilar-Salinas *et al.*	2.7 (*p* = 0.002)	2.3 (*p* = 0.014)
Harmonisation criteria (Lavie *et al*.)	5.7 (*p* < 0.001)	3.6 (*p* = 0.001)

*NCEP* National Cholesterol Education Program/Adult Treatment Panel III

Even after adjusting for BMI category, the metabolically unhealthy phenotype was associated with a higher prevalence of having HOMA-IR ≥ 2.5 for all definitions used in both sexes (Table 3 and Fig. 2). After adjusting for BMI category, the metabolically unhealthy phenotype as defined by the NCEP-2 criteria retained the strongest association with insulin resistance in females (adjusted OR of 16.1), whilst in males a metabolically unhealthy phenotype as defined by the Aguilar-Salinas *et al.* criteria retained the strongest association with insulin resistance (adjusted OR of 15.3).

## Discussion

Our data show considerable differences in the prevalence of body size phenotypes in a contemporary middle-aged population when using different diagnostic criteria. This finding is consistent with evidence from previous studies [8, 37, 38]. Our study thus reinforces the need to adopt a population-specific approach in the definition of metabolic health, as criteria developed in North European/American Caucasians might not be generalizable. Specifically, the different diagnostic criteria currently in use require reproduction and validation in specific populations to account for regional differences in genetic admixture, demographics, background prevalence of obesity and variation in anthropometric characteristics.

The prevalence of the different body size phenotypes reported in this study is markedly different from that reported by other authors. For example, when using the Aguilar-Salinas *et al*. definition of metabolic health in an Irish population, Phillips & Perry *et al.* [37] found a much lower prevalence of MHOw/O (2.2% compared to 45.2% in our cohort when using the same definition) and of MHNW (8.8% compared to 25.0% in our cohort). Direct comparison between studies is limited by population-specific differences in life-style factors, variable patient ascertainment criteria, the impact of genetic factors on adiposity and fat distribution patterns as well as temporal changes in the prevalence of body size phenotypes.

All definitions of metabolic health had a higher predictive value with respect to insulin resistance than BMI alone in both males and females. This reinforces the importance of the concept of metabolic health over a simple BMI-based classifier, which does not fully capture the underlying adverse cardiometabolic risks. Furthermore, a metabolically unhealthy phenotype was still strongly predictive of insulin resistance for all definitions used even after adjusting for BMI category in both sexes. Although, there are many studies on metabolic health, few have compared the strength of the association of metabolic health with insulin resistance when using the different definitions currently in use. Our data is important because it shows which definition has the strongest association.

This study also identifies important sex-specific effects in the ability of the various definitions of metabolic health to predict insulin resistance. A cut-off of HOMA-IR ≥ 2.5 was selected since population-based research has shown that this threshold is associated with increased mortality [34–36]. Our data shows that in females, the NCEP-2 definition demonstrated the strongest association with insulin resistance compared to other definitions. Conversely, in males, the definition by Aguilar-Salinas *et al*. had the strongest association with insulin resistance (OR of 18.7), followed by that of Lynch *et al*. and NECP-2. The Doumatey *et al*. definition also performed much better in males than in females (OR of 12.2 compared to 2.4). The NCEP-2 definition remained the best predictor of insulin resistance even after adjusting for BMI category in females.

There are possible physiological mechanisms that underlie the observed sex-specific associations. Females exhibit greater blood pressure variability than males [39]. This may be mediated by greater baroreceptor sensitivity [40] and by greater sensitivity to changes in dietary sodium [41] in females compared to males. The increased blood pressure variability in females would be expected to create greater inaccuracy in body size characterization in women, especially when using definitions which only require one abnormal criterion in order to be classified as being metabolically unhealthy (such as those by Aguilar-Salinas *et al.*, Lynch *et al.*, Doumatey *et al.*, NCEP-0, and Lavie *et al.)*. On the other hand, the NCEP-ATPIII criteria use a higher cut-off for waist circumference in males than in females. There is evidence that this cut-off may be too high in males [28]. This may explain the stronger association of the NCEP-2 definition of metabolic health with insulin resistance in females compared to males. The Aguilar-Salinas *et al.* definition uses identical cut-offs for HDL-cholesterol in males and females. In females, the HDL-cholesterol cut-off is therefore significantly lower than the one used by NCEP-ATPIII (1.0 vs 1.3 mmol/L). The former may be too low, and this may explain why the NCEP-2 definition performs better than that by Aguilar-Salinas *et al.* in females. Women are known to have higher HDL-C [42, 43]. Data from the US National Health and Nutrition Examination Survey indicates that the optimal HDL-C cut-off to predict cardiovascular disease is 1.45 mmol/L in females compared to 1.06 mmol/L in males [44]. These cut-offs are likely to differ amongst populations. For example, in Koreans the optimal HDL-C cut-offs are 1.24 mmol/L in females and 1.11 mmol/L in males [44].

The various definitions of metabolic health carry important caveats that impact on broad clinical interpretation. The metabolic health definitions currently in use are based on the findings of investigations carried out in different ethnicities. There is extensive between-study heterogeneity, with the use of different sample sizes and different gender proportions. These factors might contribute to the observed variation between males to females reported in this study. Furthermore, most definitions were not derived from studying the association of metabolic health with insulin resistance or cardiovascular disease.

An additional definition of the metabolically healthy phenotype, proposed by Meigs *et al*. is based on HOMA-IR values below the 75th centile [18]*.* In the current study*,* we did not explore this classification since we wanted to investigate which body composition phenotype is most predictive of insulin resistance without the need of determination of HOMA-IR. Furthermore, this definition results in a fixed prevalence of the metabolically unhealthy phenotype (25%) in all populations and at all times. Thus, it does not account for the dynamic nature of insulin resistance based on population-specific differences in cardiometabolic risk. The definitions proposed by Wildman *et al.* and Karelis *et al.* also incorporate HOMA-IR as one of their criteria, but they also use additional biochemical and anthropometric criteria. Hence, we calculated the prevalence rates of the various body size phenotypes when using these definitions, but we did not enter them in the logistic regression analyses to predict a high HOMA-IR.

### Strengths and limitations

The findings from this study need to be interpreted in the context of several limitations. The use of convenience sampling rather than stratified random sampling led to more females than males being recruited, which may thus explain the skewed gender ratios observed in this study. Nonetheless, the number of males recruited (190) gives a statistical power of 0.75 to detect an odds ratio of 1.75 at α = 0.05 [45]. We had 0.95 power to detect an odds ratio of 1.75 at α = 0.05 in females [45]. We used HOMA-IR as a measure of insulin resistance rather than a euglycaemic clamp, but previous studies have shown a very good correlation between the two [31, 32]. Furthermore, high HOMA-IR has been shown to be associated with increased cardiovascular, cancer and all-cause mortality [1–3]. The cross-sectional design of our study precludes drawing of definitive conclusions on long-term outcomes.

This investigation is strengthened by the use of a well-characterized and reasonably sized representative sample of middle-aged adult subjects. There are significant age-related changes in muscle mass, fat distribution and in the prevalence of body size phenotypes. Thus, the relationship between anthropometric parameters and metabolic health is likely to vary across different age groups, which should be studied separately. Anthropometric parameters were measured using standard procedures and biochemical variables were centrally analyzed under appropriate quality controls. The Maltese population consists largely of Caucasians, with other races being largely under-represented. Since there are racial differences in the relation of insulin resistance to anthropometric and biochemical parameters, we restricted our study to subjects of Caucasian ethnicity. It is therefore important that other authors replicate our findings in other ethnic groups.

## Conclusions

This study demonstrated large differences in prevalence of the various body size phenotypes when using different criteria, thereby highlighting the need for standardization of definitions. Irrespective of the definition used, the metabolically unhealthy phenotype was more strongly associated with insulin resistance than BMI category. Furthermore, a metabolically unhealthy phenotype using any definition was associated with insulin resistance even after adjusting for BMI category. This study lends further support to incorporating metabolic health in patient stratification since this offers additional information on cardiometabolic risk compared to BMI alone.

The strongest association with insulin resistance as measured by HOMA-IR in females was obtained by using the presence of >2 NCEP-ATPIII criteria to define a metabolically unhealthy phenotype, whilst in males the strongest association with insulin resistance was specified by the Aguilar-Salinas *et al.* definition. Our data therefore suggest the need for sex-specific definitions of metabolic health. Future studies should seek to replicate these findings in other age groups and populations, and to evaluate the longitudinal relationship of the different definitions to long-term cardiometabolic outcomes.

## Data Availability

The datasets used and/or analysed during the current study are not publicly available due to legal constraints but are available from the corresponding author on reasonable request.
